# Interleukin-13 reduces cardiac injury and prevents heart dysfunction in viral myocarditis via enhanced M2 macrophage polarization

**DOI:** 10.18632/oncotarget.20111

**Published:** 2017-08-10

**Authors:** Honghui Yang, Yan Chen, Chuanyu Gao

**Affiliations:** ^1^ Department of Cardiology, Zhengzhou University People's Hospital, Zhengzhou 450001, China

**Keywords:** IL-13, viral myocarditis, macrophage polarization

## Abstract

Viral myocarditis is one of the major causes of congestive heart failure and dilated cardiomyopathy. Recent reports have demonstrated an essential role of cytokines, like interleukin-13 (IL-13), in the pathogenesis of viral myocarditis, while the underlying mechanisms remain poorly defined. Here, using a coxsackie virus B3 (CVB3)-infection model in BALB/C mice, we showed that IL-13 protected mouse heart function in viral myocarditis, seemingly through reduction in T lymphocyte immunity and induction of M2 macrophage polarization. Adoptive transfer to M2 macrophages mimicked the effects of IL-13 on protection from myocarditis, suggesting that the effects of IL-13 may be primarily through regulation of macrophage polarization. Together, our data suggest that application of IL-13 treatment may reduce cardiac Injury and protect heart function in viral myocarditis via enhanced M2 macrophage polarization.

## INTRODUCTION

Some viruses, like coxsackie virus and hepatitis C virus, are known to cause viral myocarditis, as a cause for the subsequent development of dilated cardiomyopathy [1]. Recent reports have demonstrated an essential role of cytokines in the pathogenesis of viral myocarditis [2–4]. First of all, high tumor necrosis factor (TNF)-alpha, Interleukin (IL)-1-alpha and IL-1-beta were detected in the plasma of myocarditis patients with congestive heart failure [5, 6]. Moreover, granulocyte colony-stimulating factor was often upregulated in myocarditis, suggesting activation of macrophages [7–10].

These evidence of involvement of cytokines in the pathogenesis of myocarditis thus led to the approaches of using inhibitory cytokines and cytokine suppression, e.g. IL-1 receptor antagonist or IL-10 [11], or suppressing IL-4 [12], in a cytokine therapy. For example, blockade of IL-4 partially suppresses the development of experimental viral myocarditis in A/J mice, suggesting involvement of Th2 cytokines in the pathology of viral myocarditis [12]. Interestingly, IL-13 knockout (KO) mice was later found to develop enhanced experimental viral myocarditis in BALB/C mice [13]. As a pleiotropic cytokine produced by many hematopoietic cell types, IL-13 does not use the classical IL-4 receptor, but an alternative IL-4 receptor, consisting of IL-4R-alpha and IL-13 receptor alpha1 subunit [14]. Moreover, IL-13 has a decoy receptor named IL-13R-alpha2 for exerting antagonistic functions [14]. These characteristics determine a distinct function of IL-13 from IL-4 during immune responses.

Macrophages are capable of differentiating into different subtypes with a range of function in respond to various environmental cues, which is called macrophage polarization [15–17]. Macrophage M1 and M2-type responses exert the opposing activities of either killing or repairing, by which such polarized responses correspondingly induce Th1- or Th2-like responses in macrophages, respectively [15]. The typical characteristics of M1 macrophages include predominant antigen presentation, high production of IL-12 and IL-23, and high production of nitric oxide (NO) and reactive oxygen species. On the other hand, M2-type responses are mainly induced by IL-4, IL-10, or IL-13 [15]. M2 macrophages are typically characterized by the upregulation of Dectin-1, mannose receptor (CD301), scavenger receptor, CD163 and CD206. Moreover, M2 likely upregulates arginase, compared to M1 macrophages [15]. While M1 macrophages enhance Th1 response, M2 macrophages augment Th2 response, tissue remodeling and immune tolerance [15]. A coordination of a number of inflammatory modulators, signaling molecules, and transcription factors controls macrophage polarization. Activation of canonical IRF/STAT1 signaling by interferon-gamma and toll-like receptor signaling will polarize macrophages toward the M1 phenotype, while activation of IRF/STAT6 signaling by IL-4 and IL-13 will polarize macrophages toward the M2 phenotype [15]. Although these cytokines play a critical role in macrophage differentiation and polarization, its effects on viral myocarditis through macrophage polarization have not systemically studied.

In the current study, we investigated the effects of application of IL-13 treatment on cardiac Injury and protection of heart function in viral myocarditis as well as the involvement of macrophage polarization in the process.

## RESULTS

### IL-13 protects mouse heart function in coxsackie virus B3 (CVB3)-induce myocarditis

**Figure 1:**
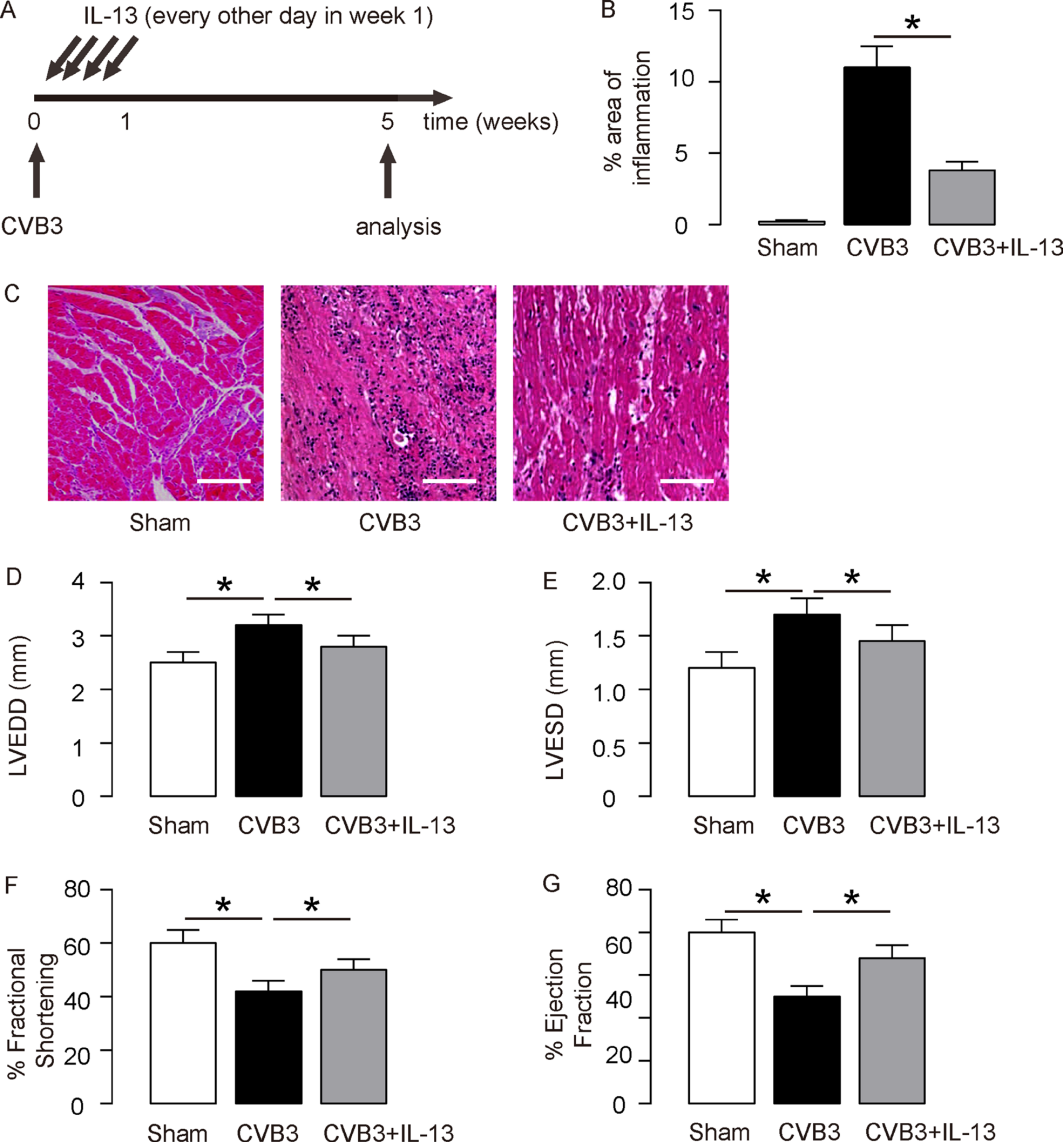
IL-13 protects mouse heart function in coxsackie virus B3 (CVB3)-induce myocarditis (**A**) We gave CVB3 to BALB/C mice, and analyzed development of viral myocarditis 5 weeks after infection. To evaluate the effects of IL-13 on myocarditis development, at the time of viral injection, we injected some mice with recombinant mouse IL-13 every other day for 3 times. Mice were divided into 3 groups: Group 1, mice were injected with saline as a control for CVB3 injection (Sham); Group 2, mice were injected with CVB3, but for control of IL-13 administration, saline of same dose and frequency was given (CVB3); Group 3: mice were injected with CVB3 and IL-13 (CVB3+IL-13). (**B**–**C**) The infection severity of the mouse heart was determined at week 5, showing by quantification (B), and by representative images (C). (**D**–**G**) Assessment of mouse heart function at week 5. (D) LVEDD. (E) LVESD. (F) % Fractional shortening. (G) % Ejection fraction. *N* = 10. ^*^p < 0.05. Scale bars are 50 µm.

We gave coxsackie virus B3 (CVB3) to BALB/C mice, and analyzed development of viral myocarditis 5 weeks after infection. To evaluate the effects of IL-13 on myocarditis development, at the time of viral injection, we injected some mice with recombinant mouse IL-13 every other day for 3 times. Mice were divided into 3 groups: Group 1, mice were injected with saline as a control for CVB3 injection (Sham); Group 2, mice were injected with CVB3, but for control of IL-13 administration, saline of same dose and frequency was given (CVB3); Group 3: mice were injected with CVB3 and IL-13 (CVB3+IL-13) (Figure 1A). At week 5, the infection of the mouse heart was determined, showing that CVB3 significantly increased heart inflammation, which was significantly attenuated by IL-13 injection, shown by quantification (Figure 1B), and by representative images (Figure 1C). The heart function of the mice from 3 groups was assessed at analysis. We found that CVB3 treatment significantly increased the left ventricular end diastolic dimension (LVEDD; Figure 1D) and left ventricular end systolic dimension (LVESD; Figure 1E) measured from a frozen M-mode tracing, and significantly reduced the percentage of Fractional shortening (% Fractional shortening; Figure 1F) and the percentage of Ejection fraction (% Ejection fraction; Figure 1G). Interestingly, IL-13 administration attenuated the detrimental effects of OVB3 on these parameters (Figure 1D–1G), suggesting that IL-13 may protect mouse heart function in CVB3-induce myocarditis.

### IL-13 reduces T lymphocyte immunity and induces M2 macrophage polarization

Next, we examined the underlying mechanisms. Since T lymphocytes are key players in the development of viral myocarditis, and since IL-13 is believed to be predominantly macrophage regulator, we used flow cytometry to examine these 2 populations in the mouse heart. First, we analyzed the percentage of CD3+ lymphocytes in mouse heart. We found that CVB3 significantly increased CD3+ lymphocytes in the mouse heart than control mice (Sham), but the increases in CD3+ cells was significantly attenuated by IL-13 injection, shown by representative flow charts (Figure 1A), and by quantification (Figure 1B). Moreover, CVB3 significantly increased F4/80+ macrophages in the mouse heart than control mice (Sham; Figure 1C-D). Interestingly, although IL-13 did not alter CD163-F4/80+ M1 macrophages in the CVB3-treated mouse heart, IL-13 significantly increased the CD163+F4/80+ M2 macrophages in the CVB3-treated mouse heart, shown by quantification (Figure 1C), and by representative flow charts (Figure 1D). Thus, IL-13 reduces T lymphocyte immunity and induces M2 macrophage polarization in CVB3-induce myocarditis.

### Preparation of IL-13-induced M2 macrophages for transplantation

In order to evaluate the role of M2 macrophages in the IL-13-attenuated CVB3-myocarditis, we isolated mouse macrophages from bone marrow of age-matched, isogeneic mice. IL-13 was given to these macrophages to induce a M2-macrophage polarization, which mimicked the *in vivo* model (Figure 3A). We found that IL-13 treatment significantly induced CD163 expression in these macrophages, suggesting M2 M2-macrophage polarization (Figure 3B). In order to confirm the change of phenotype, mRNA was isolated from the treated cells and subjected to a RT-qPCR analysis for macrophage subtype-specific genes. We found that IL-13-treated cells significantly reduced iNOS expression, and significantly increased Arginase and Declin-1 expression (Figure 3C), suggesting a M2-like polarization. These cells were thus readily used for adoptive transplantation in a gain-of-function experiment.

**Figure 2:**
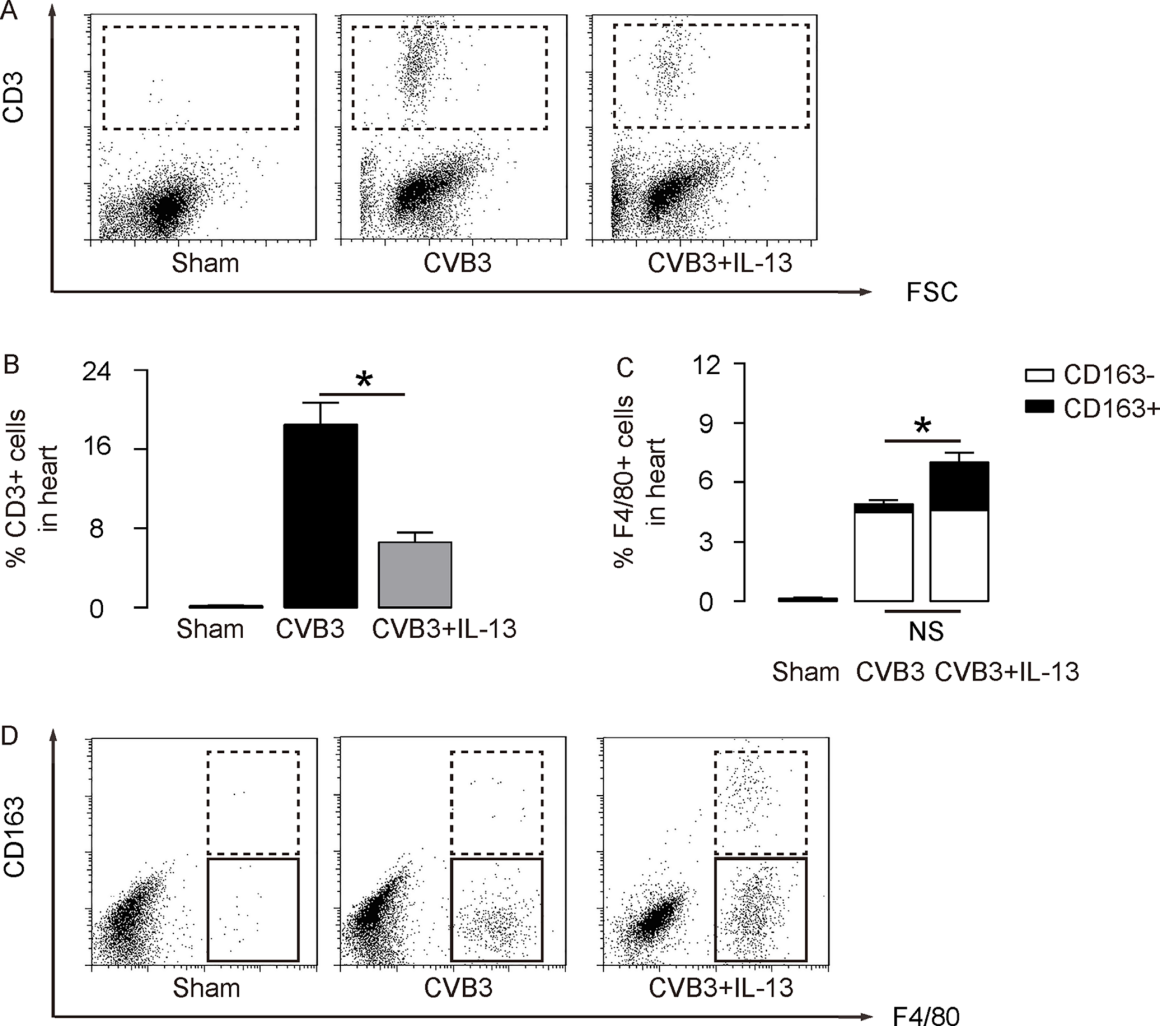
IL-13 reduces T lymphocyte immunity and induces M2 macrophage polarization (**A**–**B**) The percentage of CD3+ lymphocytes in mouse heart was assessed by flow cytometry, shown by representative flow charts (A), and by quantification (B). (**C**–**D**) The percentage of CD163-F4/80+ M1 macrophages and CD163+F4/80+ M2 macrophages in the mouse heart were assessed by flow cytometry, shown by quantification (C), and by representative flow charts (D). *N* = 10. NS: non-significant. ^*^p < 0.05.

**Figure 3:**
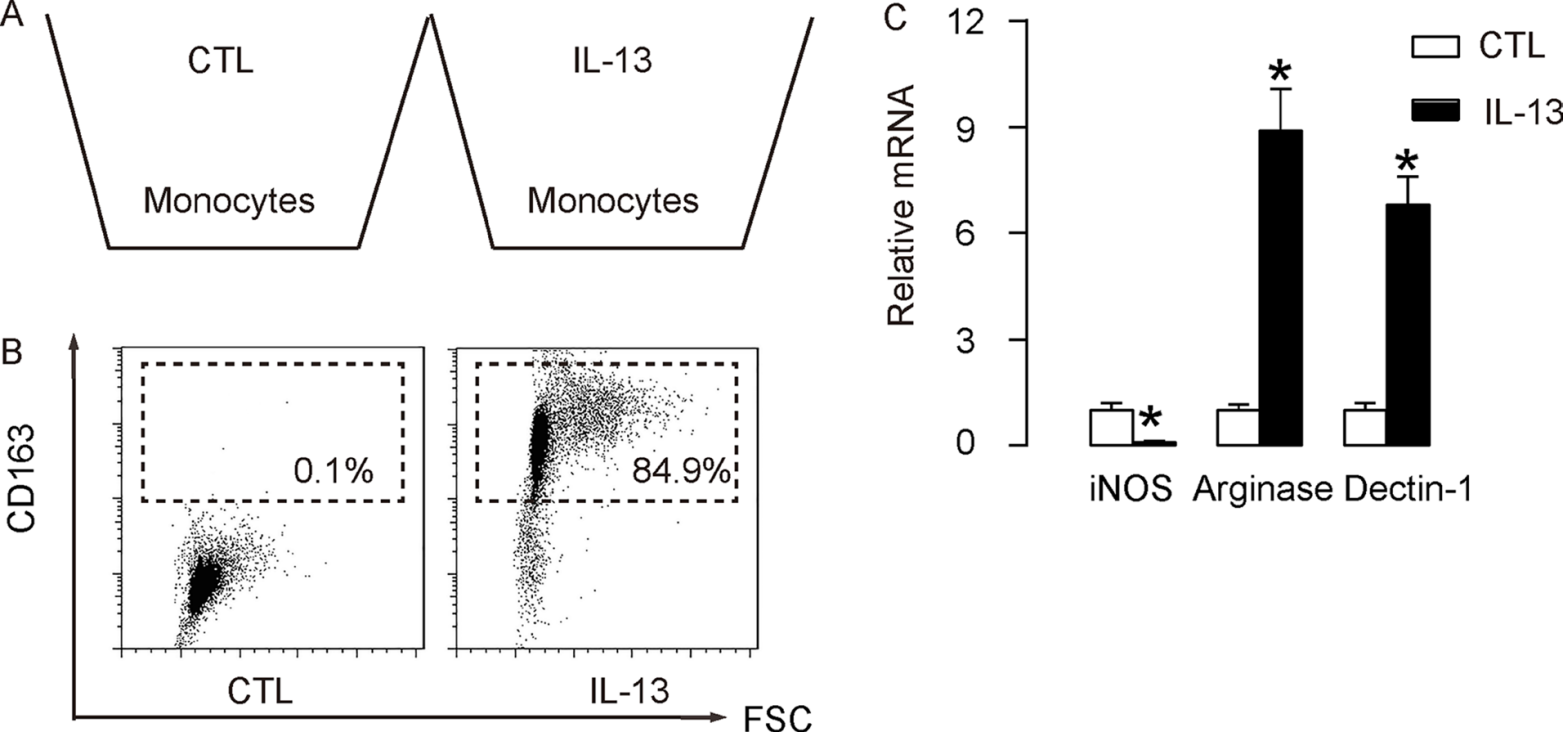
Preparation of IL-13-induced M2 macrophages for transplantation (**A**) Mouse macrophages were isolated from bone marrow of age-matched, isogeneic mice. IL-13 was given to these macrophages to induce a M2-macrophage polarization, which mimicked the *in vivo* model. (**B**) Representative flow chart for analysis of CD163 expression in IL-13-treated macrophages, compared to control (CTL). (**C**) RT-qPCR for iNOS, Arginase and Declin-1 expression in IL-13-treated macrophages, compared to CTL. *N* = 5. ^*^p < 0.05. Mφ: macrophages.

### Adoptive transfer of M2 macrophages mimics the effects of IL-13 in CVB3-myocardits

**Figure 4:**
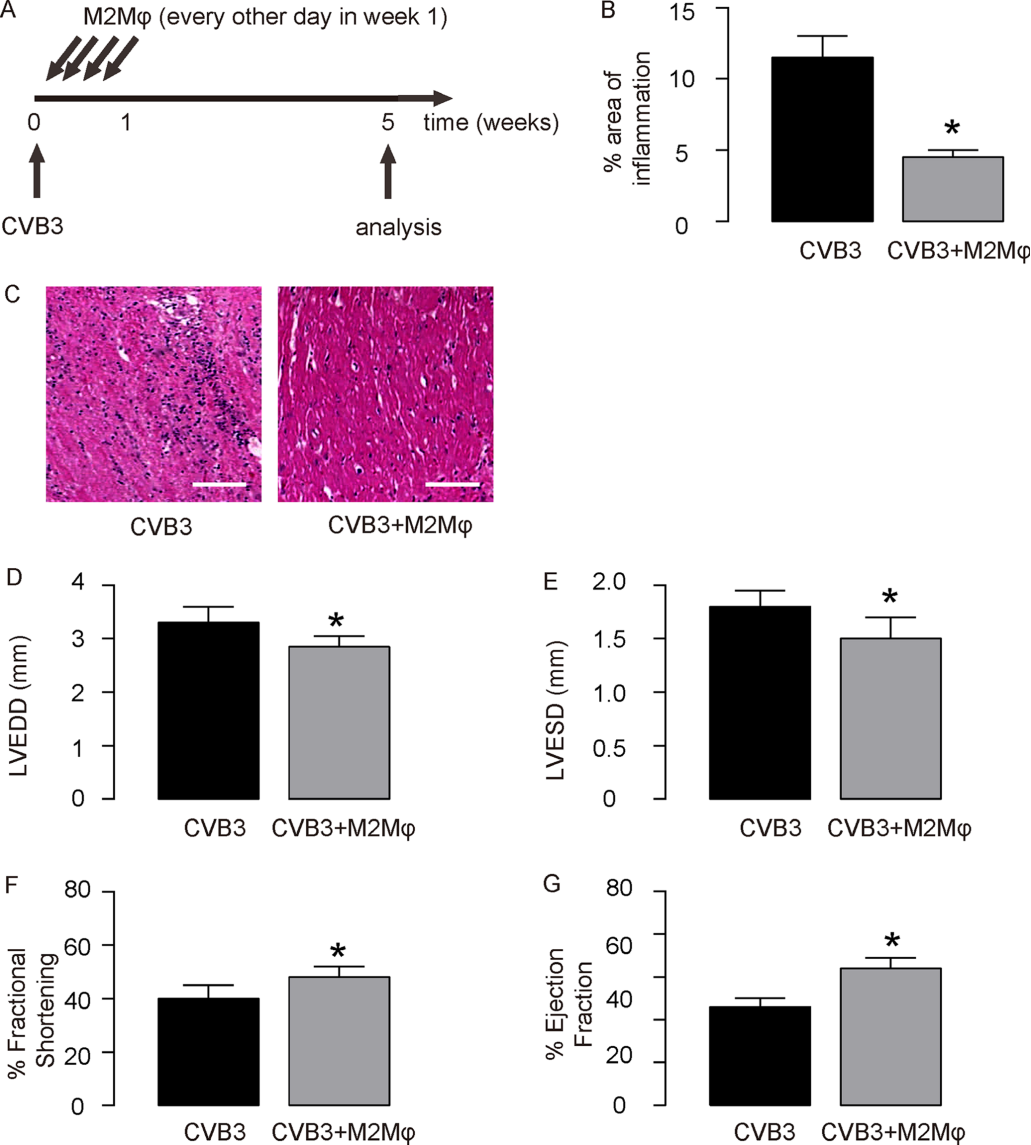
Adoptive transfer of M2 macrophages mimics the effects of IL-13 in CVB3-myocardits (**A**) Experimental design: At the time of viral injection, 10^6^ IL-13-treated macrophages were i.p. injected to some mice every other day for 3 times. The first injection was done at the time of CVB3 injection. CVB3-treated mice were divided into 2 groups. Group 1, mice were injected with CVB3, but for control of adoptive transfer of M2 macrophages, saline of same dose and frequency was given (CVB3); Group 3: mice were injected with CVB3 and received adoptive transfer of M2 macrophages (CVB3+M2Mφ). (B-C) The infection severity of the mouse heart was determined at week 5, showing by quantification (**B**), and by representative images (**C**). (**D**–**G**) Assessment of mouse heart function at week 5. (D) LVEDD. (E) LVESD. (F) % Fractional shortening. (G) % Ejection fraction. *N* = 10. ^*^p < 0.05. Scale bars are 50 µm. Mφ: macrophages.

The IL-13-treated macrophages were used for adoptive transplantation in CVB3-myocardits. At the time of viral injection, 10^6^ IL-13-treated macrophages were i.p. injected to some mice every other day for 3 times. The first injection was done at the time of CVB3 injection. CVB3-treated mice were divided into 2 groups. Group 1, mice were injected with CVB3, but for control of adoptive transfer of M2 macrophages, saline of same dose and frequency was given (CVB3); Group 3: mice were injected with CVB3 and received adoptive transfer of M2 macrophages (CVB3+M2Mφ) (Figure 4A). At week 5, the infection of the mouse heart was determined, showing that CVB3 significantly increased heart inflammation, which was significantly attenuated by adoptive transfer of M2 macrophages, shown by quantification (Figure 4B), and by representative images (Figure 4C). The heart function of the mice from 2 groups was assessed at analysis. We found that CVB3 treatment significantly increased the LVEDD (Figure 4D) and LVESD (Figure 1E), and significantly reduced the % Fractional shortening (Figure 1F) and the % Ejection fraction (Figure 1G). Like IL-13, adoptive transfer of M2 macrophages attenuated the detrimental effects of OVB3 on these parameters (Figure 4D–4G), suggesting that the effects of IL-13 on CVB3-myocarditis may be primarily through induction of M2 macrophage polarization.

## DISCUSSION

Recent reports suggest importance of cytokines in the pathology of viral myocarditis [2–4]. Moreover, approaches of inhibitory cytokine application and suppression of certain cytokine have been used in animal model for viral myocarditis [11, 12]. Unlike IL-4 [12], IL-13 knockout (KO) mice develop enhanced experimental viral myocarditis in BALB/C mice [13], but the effects of IL-13 treatment have not been examined.

The target cells of IL-4 and IL-13 are different. Although they may share IL-4R-alpha subunit, IL-13 processes different receptor subunits, IL-13R-alpha1 and IL-13R-alpha2 [14]. Since T cells do not express functional IL-13R-alpha1, IL-13 appeared to have no effects on T cells, but relatively exclusive effects on monocytes/macrophages. Hence, we believed that IL-13 may be primarily effective through macrophages in the current model, which was then confirmed in our experiments.

In contrast to interferon-gamma which specifically activates M1 macrophages, IL-13 and IL-4 are well-known to activate M2 macrophages. But when taking into concern of the specificity of target cells, modulation of IL-13, rather than IL-4, should have relative clearer effects on immunity, which results in the complete opposite effects of them on viral myocarditis [12, 13].

Compared to M1 macrophages, the role of M2 macrophages during infection and tissue injury/repair appears to be mainly suppression of immune responses and enhancement of tissue repair [15].

In our experiments, we found that IL-13 increased M2 macrophages, but did not alter M1 macrophage number. This effect resulted in alteration of the ratio of M1 versus M2 macrophages, and the alteration of growth factor secretion from macrophages, which may subsequently change the differentiation and function of T cells. Indeed, we found that the total T cells were decreased by IL-13, as a direct cause for the attenuation of the viral myocarditis. On the other hand, the unaltered number of M1 macrophages by IL-13 allowed us to use adoptive M2 macrophage transfer, rather than combination of adoptive M2 macrophage transfer and M1 macrophage elimination method, in a gain-of-function approach. The data from adoptive M2 macrophage transfer well supported the unique effects of IL-13 on macrophages, and confirmed the effects of IL-13 on heart function protection in primarily through M2 macrophage polarization.

Recently, macrophage polarization has been found out to play very critical roles in numerous diseases, e.g. tumor formation [18–21], tissue repair [22–24], and cell proliferation [20, 25–29]. Compared to adoptive transfer of M2 macrophages, *in vivo* polarization of macrophages may be a more powerful and clinic-applicable method, since macrophage polarization *in vivo* not only increases M2 macrophages, but also reduces M1 macrophages, resulting in two beneficial effects of further increases in production of trophic growth factors and removal of the detrimental effects from M1 macrophages [25, 30–32]. Recent studies have reported these approaches, e.g. used of TIPE2 [33–36]. Hence, these strategies may be tested in future studies.

To summarize, our study contributes to a clear demonstration of an IL-13-mediated protective pathway in the pathogenesis of viral myocarditis. Further dissection of the detailed cell-cell crosstalk and the underlying molecular mechanism may improve our understanding of the viral myocarditis in humans.

## MATERIALS AND METHODS

### Protocol approval and mouse housing

All methods and protocols involving mouse manipulations were approved by the Animal Care and Use Committee of Zhengzhou University People’s Hospital. Male BALB/C mice of 12 weeks of age were purchased from Shanghai Laboratory Animal Center (Shanghai, China), and maintained in the Zhengzhou University People’s Hospital conventional animal facility.

### Coxsackie virus B3 (CVB3)-induced viral myocarditis model in mice

For developing CVB3-induced myocarditis, Male BALB/C mice of 12 weeks of age were inoculated intraperitoneally with 800 plaque-forming units of a heart-passaged stock of CVB3 (Nancy strain, American Type Culture Collection, ATCC, Rockville, MD, USA), diluted in sterile phosphate-buffered saline (PBS). Recombinant mouse IL-13 (R&D Systems, Los Angeles, CA, USA) was given subcutaneously at a dose of 5 µg/mouse every other day in the first week after viral infection. The first injection was done at the time of viral injection.

### Isolation, culture and differentiation of mouse macrophages

Mouse macrophages were isolated from bone marrow, as described before [37]. Marrow from male, 12 week-old BALB/C mice was flushed out with PBS containing 20mmol/l Tris and 100mmol/l NaCl (pH 7.5) through a 23-gauge needle. Cells were pre-treated with FITC-conjugated F4/80 antibody (Becton-Dickinson Biosciences, San Jose, CA, USA) and then sorted for F4/80+ cells by flow cytometry. Purified F4/80+ macrophages were cultured in Dulbecco’s Modified Eagle Medium/F12 (DMEM/F12; Invitrogen, St. Louis, MO, USA) suppled with 10mmol/l L-glutamine, 100U/ml penicillin, 100µg/ml streptomycin and 100U/ml recombinant M-CSF (R&D Systems). For induction of macrophage polarization *in vitro* by IL-13, cultured macrophages were treated with recombinant IL-13 (R&D Systems, cat. no. 413-M) at a concentration of 20 U/ml and incubate overnight.

### Fluorescence-activated cell sorting (FACS) for leukocytes/macrophage (subtypes)

Heart tissue was minced into small pieces of 1mm in diameter, and then subjected to 0.25% Trypsine solution (Invitrogen) for 45 minutes, filtered through 45µm filter, washed 3 times with vehicle solution, and re-suspended in PBS. Cultured cells were dissociated and then analyzed directly. These single cell fractions were incubated with either PE-conjugated anti-CD3 antibody, or FITC-conjugated anti-F4/80 antibody and/or APC-conjugated anti-CD163 antibody (Becton-Dickinson Biosciences), and then sorted for leukocytes, macrophage (M1 or M2), correspondingly. Data were analyzed and quantified using Flowjo software (Flowjo LLC, Ashland, OR, USA).

### Determination of histopathological changes

Mice were evaluated for the development of CVB3-induced viral myocarditis 5 weeks after infection. Mouse Heart tissue was fixed in 10% phosphate-buffered formalin, after which paraffin embedding was performed. Longitudinal sections of 5µm-thickness were prepared for hematoxylin and eosin staining. The severity of the CVB3-induced viral myocarditis was assessed as the percentage of the heart section with inflammation compared with the overall heart section area, determined by 2 independent researchers who scored the slides separately in a blinded manner.

### Echocardiography

Trans-thoracic echocardiography was performed using the VisualSonic Vevo 660 imaging system equipped with a 40MHz transducer (VisualSonics Inc., Toronto, Canada). Ultrasonic transmission gel (Parker Laboratories, Fairfield, NJ) was applied to shaved left hemi-thorax. The mouse heart was imaged in the two-dimensional mode in the parasternal short axis view. The left ventricular end diastolic dimension (LVEDD) and left ventricular end systolic dimension (LVESD) were measured from a frozen M-mode tracing. Percentage of Fractional shortening (% Fractional shortening) is the percent change in left ventricular cavity dimensions. Percentage of Ejection fraction (% Ejection fraction) is the percentage of stroke volume to the end diastolic left ventricular volume.

### Quantitative real-time PCR (RT-qPCR)

Total RNA were extracted using the RNeasy mini kit (Qiagen, Hilden, Germany). Complementary DNA preparation and quantitative real-time PCR (RT-qPCR) were performed, using QuantiTect SYBR Green PCR Kit (Qiagen). All primers were purchased from Qiagen. Data were collected and analyzed using 2-∆∆Ct method. Values of genes were first normalized against housekeeping gene α-tubulin, and then compared to the experimental controls.

### Statistical analysis

All values represent the mean ± standard deviation (SD). Statistical analysis of group differences was carried out using a one-way analysis of variance (ANOVA) test followed by the Fisher’s Exact Test to compare two groups (GraphPad Software, Inc. La Jolla, CA, USA). A value of *p* < 0.05 was considered statistically significant after Bonferroni correction.
